# Metabolic Hallmarks of Tumor and Immune Cells in the Tumor Microenvironment

**DOI:** 10.3389/fimmu.2017.00248

**Published:** 2017-03-08

**Authors:** Kathrin Renner, Katrin Singer, Gudrun E. Koehl, Edward K. Geissler, Katrin Peter, Peter J. Siska, Marina Kreutz

**Affiliations:** ^1^Internal Medicine III, University Hospital Regensburg, Regensburg, Germany; ^2^Regensburg Center for Interventional Immunology, Regensburg, Germany; ^3^Department of Surgery, University Hospital Regensburg, Regensburg, Germany

**Keywords:** tumor metabolism, immune cell metabolism, immune escape, glycolysis and oxidative phosphorylation, cytokines, immune cell functions

## Abstract

Cytotoxic T lymphocytes and NK cells play an important role in eliminating malignant tumor cells and the number and activity of tumor-infiltrating T cells represent a good marker for tumor prognosis. Based on these findings, immunotherapy, e.g., checkpoint blockade, has received considerable attention during the last couple of years. However, for the majority of patients, immune control of their tumors is gray theory as malignant cells use effective mechanisms to outsmart the immune system. Increasing evidence suggests that changes in tumor metabolism not only ensure an effective energy supply and generation of building blocks for tumor growth but also contribute to inhibition of the antitumor response. Immunosuppression in the tumor microenvironment is often based on the mutual metabolic requirements of immune cells and tumor cells. Cytotoxic T and NK cell activation leads to an increased demand for glucose and amino acids, a well-known feature shown by tumor cells. These close metabolic interdependencies result in metabolic competition, limiting the proliferation, and effector functions of tumor-specific immune cells. Moreover, not only nutrient restriction but also tumor-driven shifts in metabolite abundance and accumulation of metabolic waste products (e.g., lactate) lead to local immunosuppression, thereby facilitating tumor progression and metastasis. In this review, we describe the metabolic interplay between immune cells and tumor cells and discuss tumor cell metabolism as a target structure for cancer therapy. Metabolic (re)education of tumor cells is not only an approach to kill tumor cells directly but could overcome metabolic immunosuppression in the tumor microenvironment and thereby facilitate immunotherapy.

## Tumor Metabolism

### Accelerated Glucose Metabolism in Tumor Cells—The “Warburg Effect”

Cells need energy to carry out their various functions. Glucose is the primary energy source for most cells and central to cell proliferation and survival. In addition, cells use lipids or amino acids such as glutamine to generate energy in form of ATP and to build biomolecules for cell growth. In non-malignant cells glucose is mainly metabolized *via* oxidative phosphorylation (OXPHOS), whereas tumor cells primarily use glycolysis for glucose metabolism, a phenomenon first described by Otto Warburg almost a century ago (1). It is clear that this metabolic alteration is important for tumor development and progression and is a hallmark of cancer (2). Vander Heiden and coauthors proposed that highly proliferating cells switch to glycolysis because mitochondria are needed as anabolic organelles for the generation of building blocks (3, 4). Accelerated glycolysis is regulated by hypoxia, oncogenes, and tumor suppressor genes, as well as kinases such as the mammalian target of rapamycin (mTOR).

Hypoxia-inducible factors (HIFs) are stabilized in response to hypoxia and induce transcription of the glucose transporter GLUT-1 and lactate dehydrogenase (LDH) (5, 6). HIF proteins are expressed in the majority of human tumors and can also be induced by the glycolytic end products pyruvate and lactate (7). HIFs also operate in conjunction with oncogenic MYC, an oncogene overexpressed in about 30% of human cancers and known to upregulate glycolytic enzymes such as LDH (8). The mTOR pathway is one of the most dysregulated signaling pathways in human cancer, leading to accelerated glucose metabolism by regulating HIF-1α and MYC (9). It was also shown that the BRAF oncogene causes upregulation of genes involved in glycolysis and its knockdown results in reduced glycolysis (10). Genetic alteration or loss of p53, one of the most frequently mutated genes in cancer, also leads to a decreased oxygen consumption and increased lactate production (11). Accordingly, tumor cells are typically characterized by increased uptake of glucose and positron emission tomography exploits this feature to identify tumors diagnostically.

Glucose is metabolized to lactate, the latter is exported from tumor cells in cotransport with protons by monocarboxylate-transporters (MCT), MCT-1 and MCT-4, which results in an accumulation of lactate lowering the pH in the tumor microenvironment (12). Gatenby and Gillies proposed that the “glycolytic phenotype” of tumor cells confers a growth advantage and is necessary for the evolution of invasive human cancers (13). This hypothesis was confirmed by Walenta et al. who found a correlation between lactate concentration in tumor tissues and the incidence of metastases, as well as a reduced overall survival in cancer patients (14).

Interestingly, tumors can display the Warburg phenotype and possess intact OXPHOS, with some cancer subtypes and cancer stem cells actually depending on mitochondrial respiration (15). Nonetheless, the “Warburg effect” is only one part of the complex tumor metabolome puzzle. Amino acid, lipid, and adenosine metabolism are also adapted to fulfill the metabolic needs of tumor cells.

### Alterations in the Key Enzymes of Lipid, Adenosine, and Amino Acid Metabolism

A considerable increase in the extracellular adenosine concentration has been reported for hypoxic tissues. Accordingly, HIF-1α has been shown to regulate the ecto-5′-nucleotidase CD73, which metabolizes adenosine monophosphate to adenosine. CD73 is expressed on the surface of tumor cells and elevated activity is found in many cancer entities (16–18). By contrast, expression of methylthioadenosine phosphorylase (MTAP), which catalyzes the conversion of 5′-deoxy-5′methylthioadenosine (MTA) to adenine and methylthioribose 1-phosphate, is reduced in many tumors including malignant melanoma (19) and hepatocellular carcinoma (20) due to either gene disruption by chromosomal rearrangement or epigenetic silencing. This results in accumulation of MTA in the tumor environment. In case of malignant melanoma, the loss of MTAP expression is linked to a higher invasive potential, leading to the hypothesis that loss of MTAP expression might contribute to metastasis of malignant melanoma (21).

Hypoxia-inducible factor also regulates genes important for lipid metabolism such as cyclooxygenase (COX)-2 (5, 22). COX enzymes are responsible for the synthesis of prostaglandins. While COX-1 is constitutively expressed in almost all tissues, its isoenzyme COX-2 is primarily found in tumors (23) and overexpression is associated with a poor prognosis in breast and ovarian cancer (24, 25). Pharmacological inhibition of COX-2 can block arginase (ARG)-1 induction in mouse lung carcinoma (26, 27) indicating that prostaglandins are important for ARG expression that hydrolyzes arginine to ornithine and urea. ARG is not only expressed in tumor cells but also in tumor-infiltrating myeloid-derived suppressor cells (MDSCs), causing depletion of arginine from the tumor environment (28). Interestingly, in myeloid cells, prostaglandins are not only involved in the regulation of ARG but also upregulate indoleamine 2,3-dioxygenase (IDO) (29), which is the rate-limiting enzyme of tryptophan catabolism through the kynurenine pathway. IDO is overexpressed in many cancers (e.g., melanoma, colon, and renal cell carcinoma) and depletes tryptophan, thus inhibiting T cell proliferation in tumor tissues (30, 31).

Glutamine is the most abundant amino acid in the body and tumors act as “glutamine traps” as high rates of glutamine uptake are characteristic for many tumor cells. The increased turnover of glutamine is in part based on the higher activity and expression of glutaminase (GLS), the first enzyme in glutamine metabolism (4, 32, 33). Accordingly, intra-tumoral glutamine levels are low, and cancer patients exhibit lowered plasma glutamine levels and conversely elevated glutamate concentrations (34).

## Immune Cell Metabolism

The metabolism of immune cells has gained increasing attention recently since it is now recognized as a sensitive factor influencing immune cell activation and differentiation. Here, we will focus on glycolytic activity and OXPHOS in immune cells.

### Glucose Metabolism in Immune Cells

Early on Otto Warburg observed increased glycolytic activity in leukocytes comparable to tumor cells; however, he interestingly attributed this phenomenon to a preparation artifact (35). It is now accepted that immune cell stimulation causes a shift toward increased glucose metabolism. In proliferating cells, the obvious reason for accelerated glycolysis is the generation of nucleotides and building blocks, e.g., *via* the connected pentose-phosphate pathway. While several studies have shown that T cell proliferation depends on glucose metabolism (36–41), this concept has been challenged recently since T cell proliferation is not affected by Ldha knockout (42). Besides supporting proliferation, glycolysis is crucial for the functional activity of immune cells. Among these effects, glycolysis has been linked to cytokine production in lymphoid and myeloid cells (42–45). While glycolysis guarantees rapid energy provision, it also participates in posttranscriptional and epigenetic control of IFNγ production (42, 43, 46).

The same signaling pathways are essential for the metabolic regulation in immune cells and tumor cells. AKT, rapidly activated upon T cell receptor (TCR) stimulation, is involved in the induction of glycolysis (47, 48) and determines expression of cytokines and adhesion molecules (47, 49). Furthermore, MYC is transiently upregulated in activated T cells (38) and increases the expression of genes related to glucose and glutamine metabolism (50). Another example is the mTOR complex that is a central regulator of immune cell metabolism and consequently differentiation of T cells into effector or regulatory phenotypes (47, 48, 51). Inhibition of T cell mTORC1 *via* rapamycin results in inhibition of glycolysis (46) and enhances CD8 memory generation (52, 53).

Not only lymphoid cells but also myeloid cells switch to glucose metabolism upon activation by a wide range of stimuli including lipopolysaccharides (54–56). The shift from OXPHOS toward glycolysis sustains ATP production, while oxygen and NADPH are available for reactive oxygen species (ROS) and nitric oxide (NO) production. ROS promotes IL-6 and TNF production and is important for bacterial defense (57–59). As a result of a truncated citrate cycle, elevated levels of citrate and succinate are detected in activated myeloid cells (60, 61). Succinate stabilizes HIF-1, which can trigger IL-1β synthesis, and accumulation of citrate serves as a precursor for lipid biosynthesis (62).

### Role of OXPHOS in Immune Cell Activation

In contrast to lipopolysaccharides, IL-4 stimulation of macrophages does not increase the glycolytic activity but rather commits these cells to OXPHOS and to increased mitochondrial biogenesis (61, 62). Besides glycolysis, OXPHOS is also immediately elevated upon anti-CD3/CD28 stimulation in T cells and supports the transition from quiescent to effector cells (41, 43, 63). OXPHOS can compensate for glucose restriction and IFNγ production is maintained (41, 64, 65). Moreover, mitochondrial ROS production synergizes with Ca^2+^ influx to activate NF-kB and AP-1 (66, 67) and is important for antigen-specific T cell activation (68). With regard to controlling immune responses, OXPHOS is particularly important for regulatory T cells (Tregs) (69) since their suppressive function is linked to glucose and lipid oxidation (70). Consistent with this general effect, FOXP3 stability is increased by acetyl-CoA and inhibition of lipid oxidation reduces FOXP3 expression and its related suppressive capacity (71). FOXP3 itself shifts metabolism toward oxidation by inhibiting AKT activation and thereby GLUT-1 expression (72). However, proliferating Tregs switch to glycolysis (73) and the induction of Tregs depends on glycolysis (74). Differing results have been published regarding the role of OXPHOS on memory T cell formation. Buck et al. have shown that mitochondrial fusion, favoring OXPHOS, is important for the generation of memory T cells (75), whereas Phan et al. have demonstrated that OXPHOS is not essential for memory T cell differentiation. The observed difference may relate to subset variations between effector memory and central memory T cells (76). In summary, it seems that T cells exhibit some metabolic flexibility and do not rely on a single energy providing pathway.

### Amino Acid Metabolism in Immune Cells

T cells, like all immune cells, are auxotroph for many amino acids and proliferation as well as activation results in an increased need for amino acids. Glutamine is essential for proliferation (38, 77, 78) during the initial growth phase as well as for protein and lipid biosynthesis in T cells (38) and for inflammasome activity, phagocytosis, and antigen expression in myeloid cells (79, 80). Glutamine supports OXPHOS, protein biosynthesis, and fuels protein glycosylation (81). Other amino acids such as arginine, tryptophan, and cysteine are also essential for T cell proliferation as well as for macrophage and MDSC function. Depletion of those amino acids by myeloid cells leads to cell cycle arrest in T cells (82–84). Arginine deprivation-induced cell cycle arrest is mediated in part by Rictor/mTORC2 which controls an amino acid-sensitive checkpoint that allows T cells to determine whether the microenvironment contains sufficient resources for proliferation (84). Elevating arginine levels induces a shift from glycolysis to OXPHOS in activated T cells and promotes the generation of central memory-like cells with enhanced survival and antitumor activity (85).

## Interplay Between Tumor and Immune Cell Metabolism

Tumor stroma consists of diverse cell populations such as T cells, NK cells, macrophages, dendritic cells, fibroblasts, and endothelial cells. Tumor-infiltrating immune cells represent a double-edged sword as they can support or inhibit tumor growth. Activated lymphoid cells can control tumor growth and malignancies, as shown in reports where dense infiltration with T cells correlates with a better prognosis (86). However, tumors often blunt the activity of tumor-infiltrating lymphocytes (87, 88) and support the differentiation of tumor-associated macrophages (TAMs) or MDSCs that promote tumor growth, e.g., by inhibiting T cells or secreting growth factors (89).

### Impact of Rapid Tumor Glucose Metabolism on Immune Cells

The Warburg effect in tumor cells may limit glucose availability and results in lactate accumulation. In renal carcinoma, we showed that accelerated glucose metabolism correlates with low CD8 T cell infiltration (90). Consistent with this finding, high glucose consumption by tumors restricts T cells in a mouse sarcoma model, leading to attenuated mTOR activity, glycolytic capacity, and IFNγ production (91, 92). However, Ho et al. demonstrated that overexpression of phosphoenolpyruvate (PEP) carboxykinase 1 could restore PEP levels and thereby improve T cell function even under glucose restriction (92).

Several studies have demonstrated that (patho)physiologically relevant concentrations of lactate modulate immune cell function *in vitro* and high levels of lactate correlate with tumor progression and metastatic spread *in vivo*. Regarding myeloid cells, lactate has been shown to inhibit monocyte activation and dendritic cell differentiation (93, 94). Furthermore, Shime et al. demonstrated that lactate increases the transcription and secretion of IL-23, a tumor-promoting cytokine involved in the generation of Th17 cells, in human monocytes/macrophages (95). Lactic acid also induces M2-polarization in TAMs *via* HIF-1α stabilization (96). Moreover, reduced Ldha expression in tumor cells resulted in diminished tumor growth and decreased the frequency of splenic MDSCs (97). Furthermore, lactate strongly inhibits the activity of antitumor effector cells such as T cells and NK cells. Husain et al. demonstrated that NK cells from Ldha-depleted tumors showed improved cytolytic function and lactate treatment of NK cells diminished their cytotoxicity. We and others have shown that proliferation and activation of human T cells is suppressed by lactic acid *in vitro* (98, 99). Treatment of T cells with lactic acid prevented TCR-triggered phosphorylation of JNK, c-Jun, p38, and NFAT activation (100, 101). Recently, our group demonstrated that human melanoma metastases exhibit a “‘Warburg phenotype’” that associated with lactate accumulation. In melanoma patients, LDHA expression correlated with T cell activity and LDHA-associated lactic acid production and acidification impaired IFNγ expression in tumor-infiltrating T cells and NK cells, thereby inhibiting tumor immunosurveillance and promoting tumor growth (101). Increasing evidence supports the view of an immunosuppressive effect of rapid glucose metabolism on development of tumor immunity.

### Changes in Tumor Amino Acid and Adenosine Metabolism Suppress T Cell Function

Tumor cells and activated immune cells require a continuous supply of amino acids such as tryptophan, arginine, and glutamine for anabolic macromolecule synthesis. A metabolic competition between tumor cells and immune cells can therefore lead to nutrient deprivation. Regarding availability of tryptophan, IDO is overexpressed in many cancers and IDO-expressing tumor cells are not rejected by specific T cells (30). Accordingly, in colorectal cancer, IDO expression is associated with low T cell infiltration and reduced survival (102). IDO-expressing tumor cells secrete tryptophan metabolites like kynurenines, suppressing cytotoxic effector functions *via* downregulation of TCR CD3 ζ-chain and induce FOXP3^+^ Treg differentiation (103). In line, upregulation of IDO is associated with a high infiltration of FOXP3^+^ cells in thyroid carcinoma (104). Arginine depletion occurs in ARG or NO synthase overexpressing tumors, subsequently leading to unresponsive T cells (105). Furthermore, arginine deprivation by tumor-infiltrating MDSCs impairs T cell function (106). Interestingly, arginine depletion not only blunts the antitumor T cell responses but can also induce MDSC generation *in vivo* (107). Moreover, MDSCs resembling tumor-associated M2 macrophages rely on glutamine metabolism, whereas M1 macrophages are characterized by increased glycolytic flux (60). Glutamine deprivation promotes Treg generation (108) and often results in glutamate accumulation, which in turn suppresses T cell activity (109, 110). Therefore, the balance of amino acids within a tumor has substantial effects on the development of the local immune response.

CD73 expression on tumor cells results in adenosine accumulation in the tumor microenvironment that inhibits activation and cytotoxic capacity of T and NK cells (111, 112). Besides T cell inhibition, adenosine has a positive impact on myeloid cells. Adenosine-generating mouse Lewis lung carcinoma cells attract myeloid cells that differentiate into TAMs, which promote tumor growth (113). Additionally, we could show that MTA suppresses antigen-specific T cell proliferation, activation, and cytokine production *via* inhibition of AKT and protein methylation (114). CD73 and COX-2 both are regulated by hypoxia and HIF. Tumor cells frequently display increased COX-2 activity and prostaglandin secretion, thus suppressing T cell function and inducing MDSCs (115). Other lipids such as gangliosides are synthesized and shed by tumor cells, especially under hypoxia. Circulating gangliosides have been shown to suppress T cell function thereby contributing to immunosuppression (116). However, a competition for lipids between tumor cells and immune cells as shown for glucose has not been reported so far, thus immune cell function should not be restricted due to limited fatty acid availability.

## Targeting Tumor Metabolism

Alterations in tumor cell metabolism represent attractive targets for the development of anticancer drugs. However, targeting tumor cell metabolism may also harm immune cell functions that contribute to tumor elimination. This influence on targeting overlapping metabolic requirements of tumor and immune cells needs to be considered especially when immunotherapy is combined with antimetabolic drugs.

### Targeting the “Warburg Effect” and Mitochondrial Activity

Early after Warburg’s observation that tumor cells show major differences in glucose metabolism compared to non-malignant cells, some attempts focused on the inhibition of tumor glucose metabolism for cancer treatment (117). These studies used 2-deoxyglucose (2-DG), a non-metabolizable glucose analog and inhibitor of hexokinase, the enzyme that catalyzes the initial step of glycolysis. This approach has regained attention during the last years (118, 119) and new drugs have been developed such as the hexokinase inhibitor 3-bromopyruvate (120). We and others have shown that inhibition of glycolysis by 2-DG sensitizes acute lymphoblastic leukemia cells to glucocorticoids (121, 122). 2-DG severely disturbs T cell proliferation and activation, although its effects may reach beyond glycolysis inhibition (41, 46). These results suggest that anti-glycolytic drugs should inhibit T cell function. Surprisingly, deletion of Ldha in T cells did not appreciably affect proliferation and growth, but did reduce IFNγ production (42). Therefore, immunological “side effects” of LDH inhibitors like that of the small-molecule inhibitor FX11 or Galloflavin should be considered when administered for tumor therapy (123, 124).

Conflicting results have been reported regarding dichloroacetate (DCA), which induces a shift from glycolysis to OXPHOS and inhibits growth of tumor cells *in vitro* and in murine tumor models. DCA was found to synergize with 2-DG in complex IV deficient cells (125), whereas other researchers have demonstrated that it suppresses apoptosis induction by cisplatin and doxorubicin. Unfortunately, DCA is not tumor cell specific, therefore, the same shift in glucose metabolism occurs in immune cells, leading to induction of FOXP3^+^ Tregs (126).

Targeting the lactate transporters MCT-1 to -4 represents another approach to overcome the “Warburg effect” in cancer cells. The second-generation MCT-1/MCT-2 inhibitor (AZD3965) is currently in phase I clinical trials for advanced solid tumors and diffuse large B cell lymphomas (http://www.clinicaltrials.gov/ct2/show/NCT01791595). However, inhibition of MCT-1/-2 also reduces T cell proliferation (127). Recently, Eichner et al. described that thalidomide, lenalidomide, and pomalidomide destabilize the CD147–MCT-1 complex that results in a loss of cell surface expression of MCT-1 (128). However, MCT-1 suppression may be of limited efficacy as many tumor cells express not only MCT-1 but also MCT-4. Lenalidomide has been shown to promote IL-2 expression in T cells (129), raising the possibility that application of lenalidomide could suppress tumor cell proliferation without affecting T cells.

We have investigated the effect of diclofenac, a non-steroidal anti-inflammatory drug, on glucose metabolism and showed that diclofenac is taken up by tumor cells and interferes with lactate secretion (130). Recently, the impact of diclofenac on lactate transport was confirmed in MCT-expressing oocytes (131). In a glioma model, diclofenac lowered lactate levels, decreased tumor growth, and tumor-infiltrating dendritic cells regained their capacity to produce IL-12. Moreover, diclofenac reduced the number of tumor-infiltrating Tregs (132). Application of diclofenac should therefore be feasible even in combination with immunotherapies.

A well-known master regulator of tumor and immune cell metabolism is mTOR. Analogs of rapamycin, an immunosuppressive drug, have been approved for treatment of some cancers based on direct effects on tumor cell proliferation, glycolysis and inhibition of angiogenesis (133, 134). From another perspective, however, treatment with rapamycin reduces the proliferation of effector T cells and stabilizes/expands Tregs (135, 136), but at the same time can increase the presence of antitumor CD8 effector memory cells (137, 138). Therefore, mTOR inhibition has both potentially positive and negative effects on tumor immunity, which are worthy of further investigation. Interestingly, these dual properties of immunosuppression and immune activation may be taken advantage of in the setting of posttransplantation malignancies that plague organ transplant recipients (139).

Besides glycolysis, OXPHOS is also a possible target structure in cancer cells. Several reports have described anticancer effects of biguanides, such as the diabetes therapeutics metformin and phenformin, which are known to inhibit the mitochondrial complex I. Interestingly, those effects seem to be partially immune-mediated as metformin improved T cell function *in vivo* (140). Furthermore, sorafenib limits respiration in tumor cells and concomitantly decreases Treg numbers in patients (141). Further investigations along this line should prove to be informative.

### Direct and Indirect Targeting of Amino Acid Metabolism

The dependency of tumor cells on extracellular arginine led to the development of arginine-depleting drugs, most prominently ADI-PEG20 (142). However, arginine depletion is clearly a double-edged sword in tumor immunology as arginine availability is crucial for proper T cell function. The same holds true for the application of GLS inhibitors. Such drugs might not only affect tumor cells but also impede T cell function. As arginine, tryptophan, and glutamine are essential for T cell function, it might be more appropriate to prevent amino acid depletion by tumor cells or myeloid cells instead of reducing amino acid levels. This approach is currently tested in a clinical trial with CB-1158, an ARG inhibitor, in combination with checkpoint therapy (NCT02903914).

In line, pharmacological inhibition of COX-2 blocks ARG-1 expression in MDSC and prevents the local and systemic expansion of MDSCs, leading to a lymphocyte-mediated antitumor response (26, 27). Immunotherapeutic approaches might therefore benefit from a concurrent blockade of COX-2 activity (143). Moreover, COX is involved in the upregulation of IDO expression in myeloid cells (29). Therefore, pharmacological inhibition of COX could also reduce tryptophan depletion by IDO-expressing tumor and tumor-infiltrating cells. Direct targeting of IDO with siRNA promoted antitumor immunity *in vivo* in a murine bladder tumor model (144) and IDO-silenced dendritic cells enhanced tumor antigen-specific T cell proliferation, cytotoxic activity, and decreased Treg numbers (145). Drugs targeting this pathway are already in clinical trials with the aim to revert cancer-induced immunosuppression (146).

### Combination of Antimetabolic Targeting with Immunotherapy

Clinical benefits from immune-checkpoint inhibition are still modest due to the tumor microenvironment facilitating immune escape. Therefore, an immune- and antimetabolic combination treatment could be a promising strategy. Allard et al. demonstrated that targeted blockade of CD73 significantly enhances the therapeutic activity of anti-PD-1 and anti-CTLA-4 monoclonal antibodies (147). In line, Zelenay and colleagues showed that combination of COX-1 and COX-2 inhibitors with checkpoint blockade immunotherapy can result in melanoma eradication (148). Combining these basic forms of therapy holds real promise for the future.

## Summary and Conclusion

**Figure 1:**
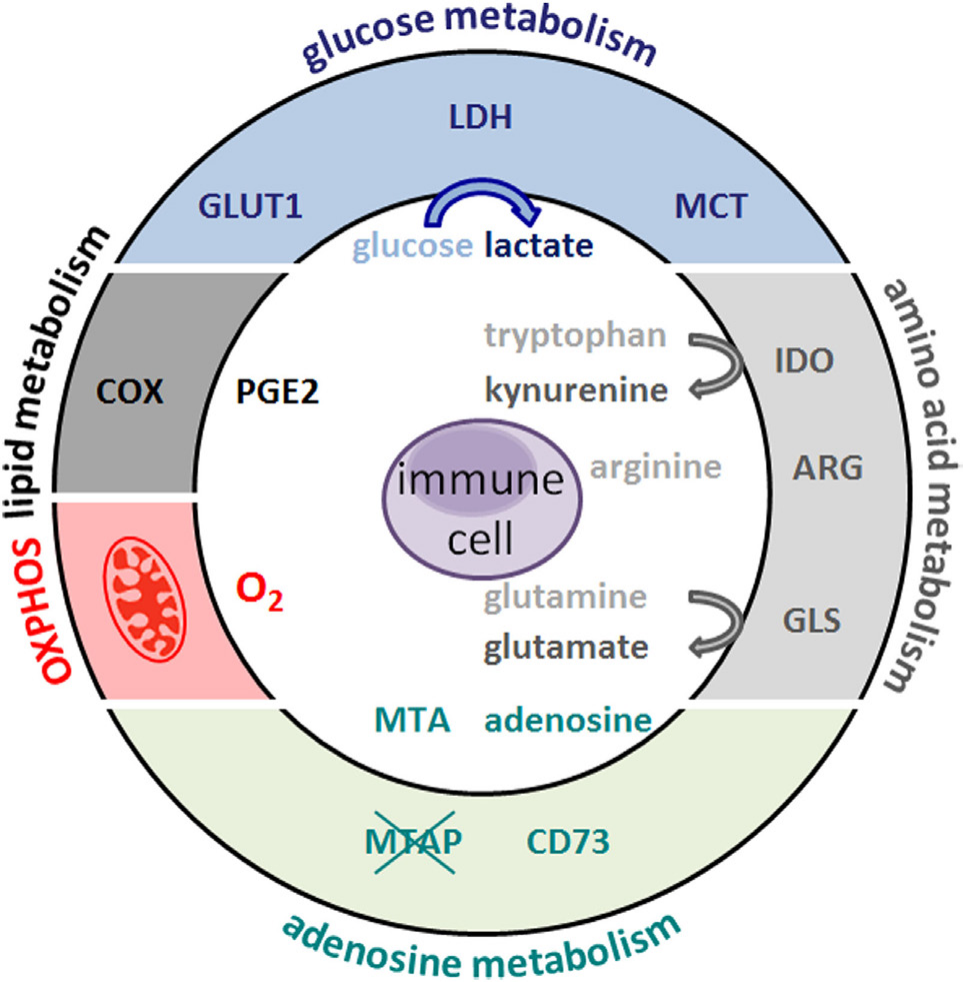
**Metabolic hallmarks of tumor cells and the interplay between tumor cells and immune cells**. Tumor cells exhibit high expression of glucose transporteres (GLUT), lactate dehydrogenase (LDH), cyclooxygenase (COX), arginase (ARG), indolamine 2,3-dioxygenase (IDO), glutaminase (GLS), and oxidative phosphorylation (OXPHOS). As a consequence, glucose and the amino acids arginine, tryptophan, and glutamine are depleted from the tumor microenvironment and nutrient restriction leads to an anergic status of antitumoral cytotoxic T cells. In addition, accelerated glycolysis by tumor cells results in lactate production and secretion *via* monocarboxylate-transporters (MCT). Lactate and other metabolites, such as glutamate, prostaglandins (PGE2), and kynurenines, affect immune cells. Overexpression of the ecto-5′-nucleotidase (CD73) leads to adenosine formation; loss of methylthioadenosine phosphorylase (MTAP) results in methylthioadenosine (MTA) accumulation in the tumor environment.

The antitumor immune response is not only suppressed by an altered tumor metabolism but also by the metabolism of tumor-associated cell populations. Tumor cells and immunoregulatory myeloid cells such as MDSCs deprive neighboring cells of essential amino acids or sugars thus removing fuels for antitumor immunity. In addition, accumulation of “waste products” such as lactate, glutamate, PGE2, or kynurenines further limit lymphoid antitumor effector functions (Figure 1).

**Figure 2:**
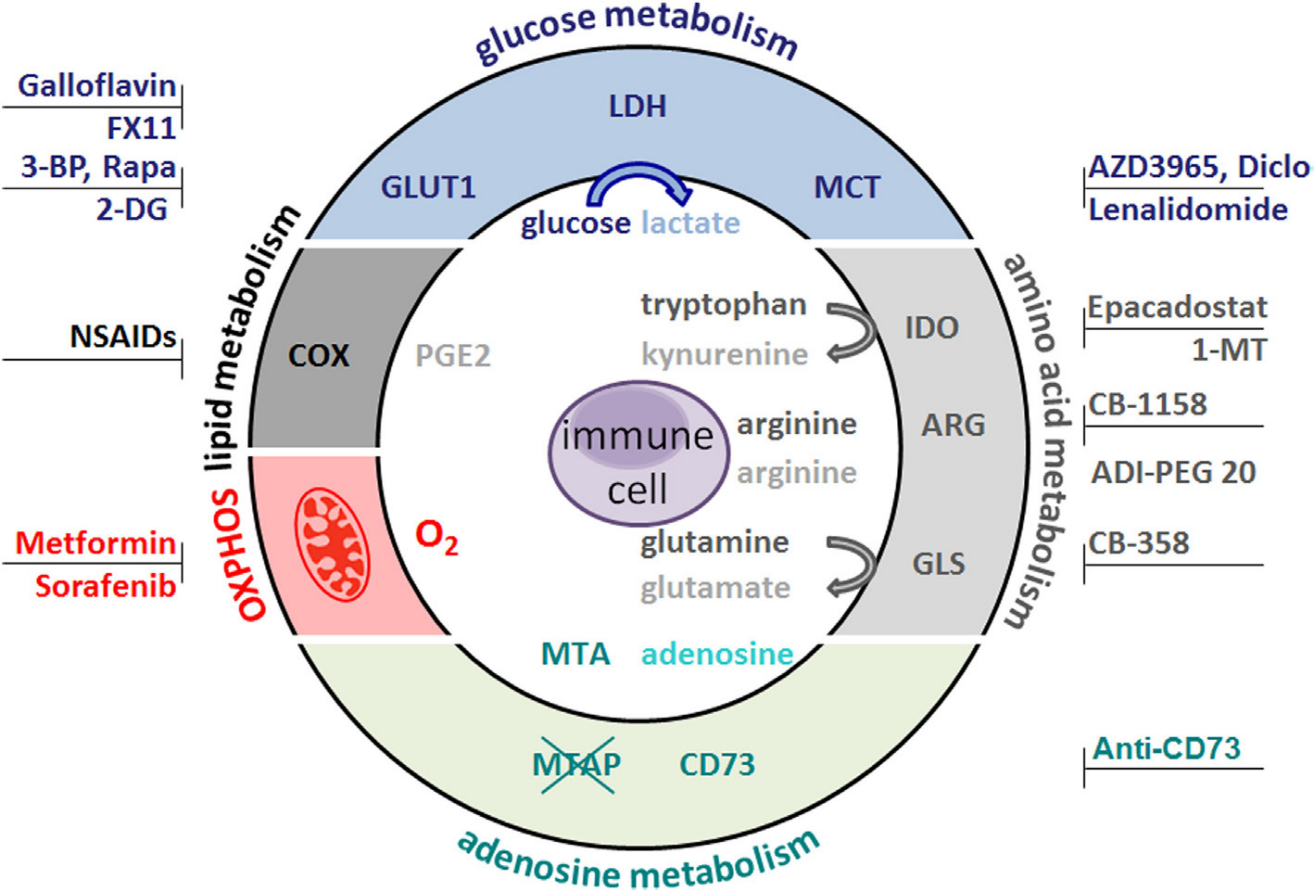
**Metabolic target structures in tumor cells and possible inhibitors**. Glucose metabolism is an attractive target for cancer therapy. Rapamycin (Rapa) inhibits the mammalian target of rapamycin pathway and glycolysis. 2-Deoxyglucose (2-DG) and bromopyruvate (3-BP) target hexokinase II, the rate-limiting enzyme of the glycolytic pathway. The lactate dehydrogenase (LDH) inhibitors FX11 and galloflavin block lactate production. Diclofenac (Diclo), lenalidomide, and AZD3965 limit lactate secretion *via* blocking lactate transporters (MCT). Oxidative phosphorylation (OXPHOS) is diminished by metformin and sorafenib. CB-389 is a glutaminase (GLS) inhibitor elevating glutamine levels while concomittantly decreasing glutamate. CB-1158 inhibits arginase (ARG) and thereby increases arginine levels, whereas ADI-PEG20 depletes arginine. Antibodies against the ecto-5′-nucleotidase (CD73) inhibit adenosine formation. Non-steroidal anti-inflammatory drugs (NSAIDs) block cyclooxygenase (COX) activity and decrease prostaglandin (PGE2) production. Indolamine 2,3-dioxygenase (IDO) can be targeted by Epacadostat and 1-methyl-tryptophan (1-MT), which results in lower kynurenine secretion and higher tryptophan levels.

Several therapeutic strategies aim to target tumor metabolism (Figure 2). However, stimulated immune cells with antitumor potential are known to be metabolically active and thus potentially sensitive to metabolic modulation. Therefore, pharmacological strategies should optimally target metabolic pathways that are differently utilized by pro-tumor and antitumor cell populations. This approach is exemplified by the effect of COX inhibitors, where they re-educate MDSCs by decreasing expression of amino acid-depleting enzymes such as IDO or ARG, thus releasing the brake on antitumor responses of T cells that rely on tryptophan and arginine abundance. Another example of a fine-tuned metabolic intervention could be pharmacological reduction of lactate efflux leading to decreased tumor cell proliferation, while supporting antitumor immune responses. Boosting antitumor immune function by antimetabolic treatments could also increase the efficacy of immunotherapies such as checkpoint blockade and may represent future avenues in the treatment of cancer patients.

## Author Contributions

KR, KS, EG, GK, KP, PS, and MK have designed the figures and wrote the manuscript.

## Conflict of Interest Statement

The authors declare that the research was conducted in the absence of any commercial or financial relationships that could be construed as a potential conflict of interest. The reviewer AZ and handling editor declared their shared affiliation, and the handling editor states that the process nevertheless met the standards of a fair and objective review.
